# Long-term Mechanical Durability of Inspiris RESILIA Surgical Aortic Valves

**DOI:** 10.1016/j.atssr.2024.11.013

**Published:** 2024-12-16

**Authors:** Sanchita S. Bhat, Jae Hyun Kim, Shweta Karnik, Maryam Bagheri, Lakshmi Prasad Dasi

**Affiliations:** 1 Wallace H. Coulter Department of Biomedical Engineering, Georgia Institute of Technology and Emory University, Atlanta, Georgia

## Abstract

**Background:**

Prolonged mechanical durability and hemodynamic characteristics after durability tests of surgical valves need to be studied. Inspiris RESILIA (Edwards Lifesciences), a relatively recent and novel surgical aortic valve, was evaluated after accelerated wear testing.

**Methods:**

Three 21-mm and 3 23-mm Inspiris valves were cycled in the durability tester for 2 billion cycles (equivalent to 50 years) and were compared with a control valve of respective size. All valves were subjected to analysis, including particle image velocimetry for downstream flow fields and *en face* imaging for geometric orifice area. Valves were tested according to International Standards Organization 5840 protocols.

**Results:**

All tested valves met the International Standards Organization durability requirements. The mean effective orifice areas for the 21-mm Inspiris control and test valves were 1.82 cm^2^ and 2.04 ± 0.01 cm^2^, respectively. The mean effective orifice areas for the 23-mm Inspiris control and test valves were 2.13 cm^2^ and 2.41 ± 0.16 cm^2^, respectively. Hemodynamics, leaflet kinematics, and local flow fields of the test valves all remained consistent with those of the control valves.

**Conclusions:**

Inspiris RESILIA surgical aortic valves demonstrated very good hemodynamic performance after an equivalent of 50 years of simulated in vitro durability. In the absence of biologic agents, the valves showed no signs of gross damage. The Inspiris RESILIA showed a performance similar to that of 1 billion cycled valves from our previous study.

**In Short**

▪ Inspiris RESILIA surgical aortic valves sizes 21 mm and 23 mm were subjected to accelerated wear testing for 2 billion cycles, equivalent of 50 years, in the accelerated wear tester.
▪ Two billon cycled test valves maintained hemodynamic stability and showed consistent leaflet kinematics, geometric orifice areas, and peak downstream velocities compared with 0 cycled and 1 billion cycled valves.

Aortic stenosis is the most common form of valvular disease, and its prevalence increases with age.^1^ The 2020 guidelines of the American Heart Association and the American College of Cardiology recommend surgical aortic valve replacement (SAVR) for patients aged <65 years.^2^ Several studies have elucidated the failure of SAVRs over time, mainly from structural valve deterioration (SVD) in response to morphologic and hemodynamic changes.^3^ Hemodynamic SVD can be characterized by cardiac systemic functional changes, usually diagnosed with echocardiography. Conversely, morphologic SVD is characterized by leaflet failure (eg, pathologic thickening, calcification) and loss of leaflet integrity and leaflet function. Available data suggest that SVD usually becomes concerning and clinically significant after the first 10 years.^4^

Failure of SAVRs as a result of morphologic changes such as leaflet calcification and failure have been linked to material changes and chemical instabilities, specifically the release of free aldehydes.^5^ A relatively new SAVR valve, the Inspiris RESILIA aortic valve (Edwards Lifesciences), which is based on the Perimount valve design, has been developed to alleviate some of these issues with its novelty in tissue treatment and expandable stent frame. The RESILIA treatment is a novel integrity preservation proprietary technology that includes stable capping of free aldehydes, thus preventing calcium binding, and glycerolization, which further prevents exposure to aldehydes and preserves the leaflets to reduce calcification. Studies have shown that this approach has advantages, particularly to avoid the adverse effects of SVD over time.^6^^,^^7^ Clinically, the Inspiris RESILIA valve has shown a good safety profile, excellent hemodynamics, and performance comparable to that of commercially available SAVRs.^8^^,^^9^

Even before clinical use, understanding the valve’s mechanical durability is paramount to validate the performance and observe any bulk hemodynamic changes in simulated environments. As such, the International Organization for Standardization (ISO) requires bioprosthetic heart valves to be tested to a minimum of 200 million cycles (equivalent to 5 years in vivo) as specified in ISO 5840-2:2021, to establish their performance and long-term mechanical durability. The Inspiris RESILIA valve was recently tested to 1 billion cycles (equivalent to 25 years in vivo) and demonstrated good in vitro hemodynamic and downstream characteristics after long-term durability testing.^10^ Although long-term durability was established, this study investigates changes in flow dynamics, if any, after further testing of the long-term mechanical durability of Inspiris RESILIA valves (tested to 2 billion cardiac cycles, equivalent to 50 years in vivo) compared with control (0 cycled) valves of the same size.

## Material and Methods

### Valve Selection

Three 21-mm and 3 23-mm Inspiris surgical aortic valves were chosen for the accelerated wear tester and hemodynamic tests. Hemodynamic variables were measured for each valve at both control (0 cycled) and 2 billion cycles (after accelerated wear testing [AWT]). A separate uncycled valve of each size served as a “control” for particle image velocimetry (PIV) and leaflet kinematic measurements. A total of 8 valves were tested for this study.

### Accelerated Wear Testing

An accelerated wear tester was used to evaluate valve fatigue time by accelerating the beat rate. Valve testing was performed in saline solution at room temperature,^10^ as detailed in ISO 5840:2021. The valves ran for a total of 10 years in real time, which equals 2 billion cardiac cycles (equivalent to 50 years in vivo). The peak differential closing pressure was maintained at >100 mm Hg for at least 5% of each pressure cycle through the testing duration. The average peak closing pressure for each valve was close to 120 mm Hg. Additional details on AWT procedures are given in the previous durability study.^10^

### In Vitro Pulsatile Flow Loop Left Heart Simulator

The leaflet kinematic evaluations were conducted in the Georgia Tech Left Heart Simulator, a validated pulsatile flow loop that simulates physiologic and pathophysiologic conditions of the heart.^10^ The valves were mounted in an aortic chamber, which was an idealized rigid acrylic chamber. The schematic of the in vitro flow loop is shown in Supplemental Figure 1. Flow conditions used were described previously.^10^

### High Speed En Face Leaflet Kinematics and Particle Image Velocimetry

Leaflet kinematics or the quantification of leaflet opening may be studied using en face images of valve opening and closure. An instantaneous geometric orifice area (GOA) can be derived during systole. Oscillations in GOA may indicate leaflet flutter and could influence the local flow patterns surrounding the leaflet.

To examine the kinematics of the leaflets and to provide data for leaflet modeling, en face high-speed videos were recorded during experiments for both valves and for both orientations. The leaflets were illuminated by light-emitting diode work lamps, and the images were recorded by a high-speed monochromatic camera (Nikon ED AF Micro NIKKOR, 200-mm lens) at 1000 frames per second. In addition to observing the leaflet kinematics qualitatively, we also used the still en face videos at peak systole to calculate the GOA. A custom algorithmic mask was used to calculate the GOA for each frame and then was integrated along the cardiac cycle to obtain a GOA integral value.

PIV analysis was conducted for all valves to examine the local flow fields. The details of the PIV setup and data analysis are shown in our previous report^10^ and in the Supplemental Methods. A schematic is shown in Supplemental Figure 2.

## Results

The data presented are taken from a single test valve chosen at random because all valves of the same size group had similar downstream flow features.

### Accelerated Wear Testing and Hemodynamic Performance

The hemodynamic data for the study valves are provided in the Table. The valves exceeded the minimum effective orifice area (EOA) acceptance criteria after enduring 2 billion cycles of AWT. Moreover, the average regurgitant fraction for 2 billion cycle valves was lower than the ISO 5840-2:2021 regurgitant fraction of 10%.

**Table:** Hemodynamic Results

Valve Size	Valve Type	EOA, cm^2^	Total RF, %	Mean TVPG, mm Hg
21	Inspiris RESILIA			
	Control (n = 1)	1.82	2.42	10.44
	Test (n = 3)	2.04 ± 0.01	2.74 ± 0.45	13.23 ± 0.23
23	Inspiris RESILIA			
	Control (n = 1)	2.13	2.8	8.33
	Test (n = 3)	2.41 ± 0.16	3.15 ± 0.30	9.19 ± 0.88

EOA, effective orifice area; RF, regurgitant fraction; TVPG, transvalvular pressure gradient.

### Leaflet Kinematics

**Figure 1:**
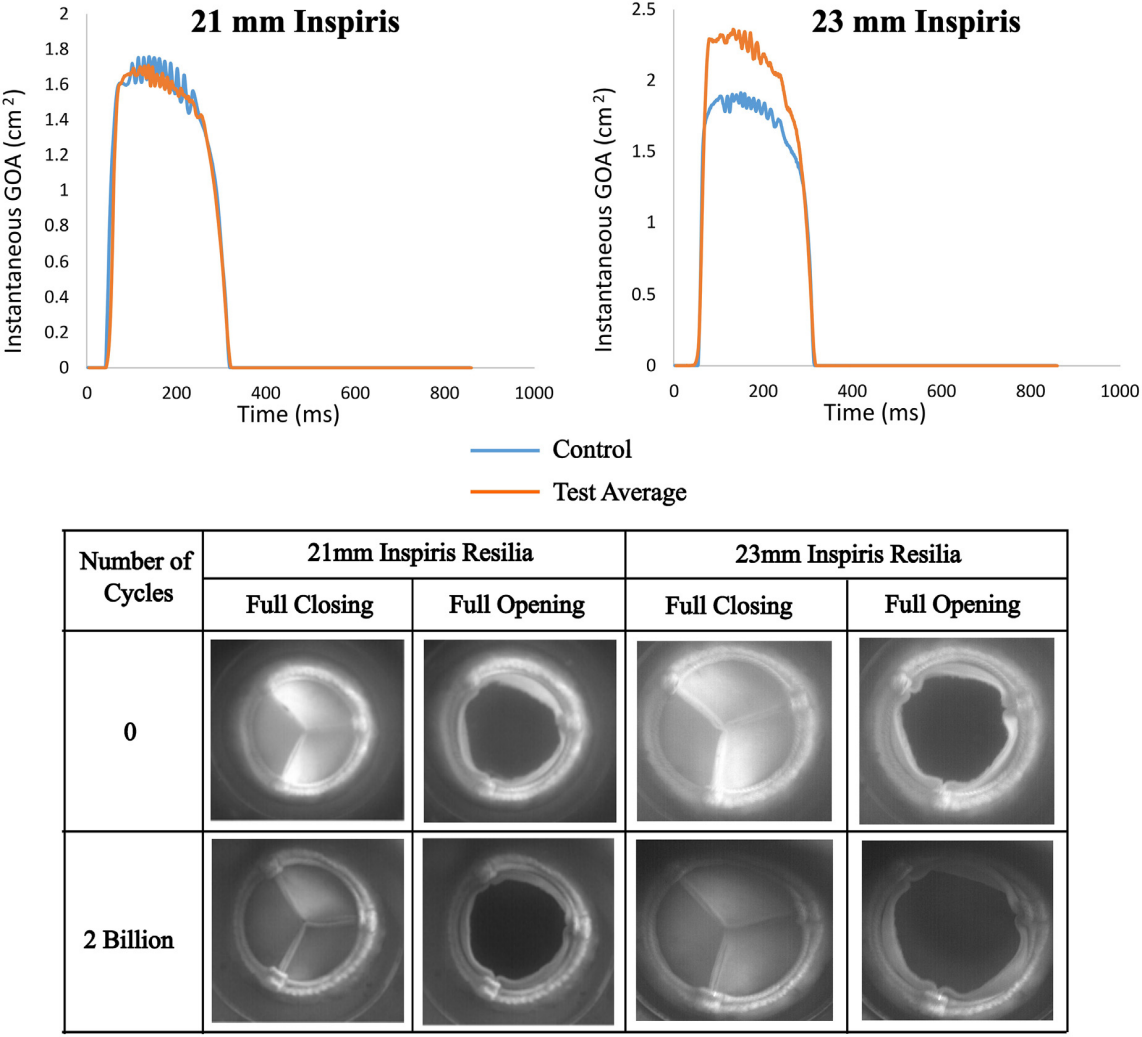
Instantaneous geometric orifice area (GOA) and en face image comparison between the control valve and the test Inspiris valve (Edwards Lifesciences).

Representative images of fully open and fully closed control and test valves and instantaneous GOA are shown in Figure 1. No tears or abrasions were observed in control or test valves.

Overall, the leaflet kinematics assessment showed that the GOA remained similar. For 21-mm Inspiris RESILIA valves, GOA magnitudes remained similar between test and control groups. During the systolic phase, leaflet flutter was observed in all valves. Additionally, despite changes in magnitude, the valves opened and closed at the same time, with no changes in overall leaflet behavior .Comparing the GOA integral for all groups, as shown in Supplemental Table E, we see a reduction in overall GOA for the 23-mm Inspiris RESILIA valves from control to 2 billion cycles. We see a slight increase in GOA for the 21-mm Inspiris RESILIA valves. Video 1 is an en face high-speed video of an Inspiris RESILIA 21-mm valve.

### Local Flow Characteristics

**Figure 2:**
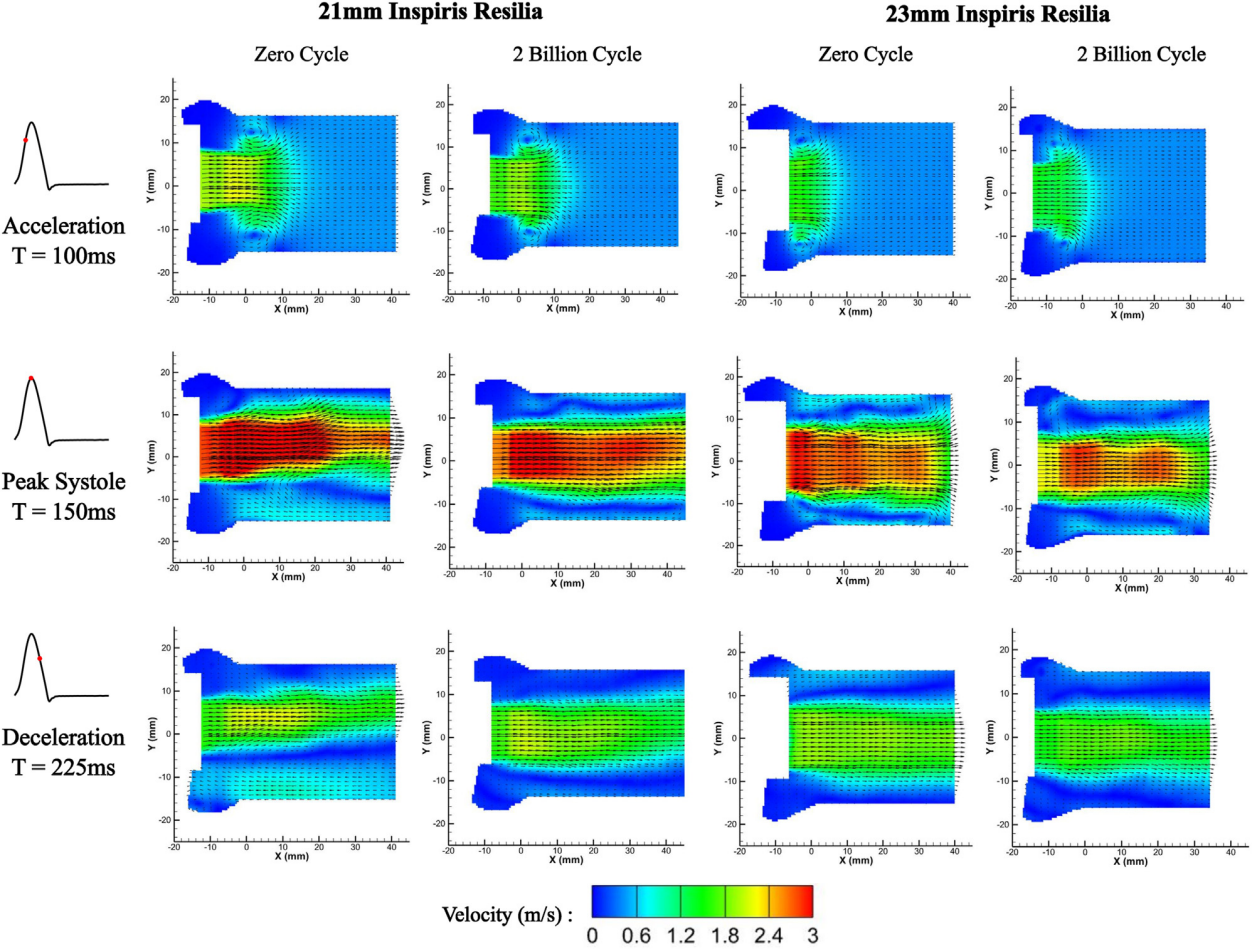
Comparison in the downstream flow fields between the control valve and the test Inspiris valve (Edwards Lifesciences).

The downstream flow characteristics from acquired PIV images are shown in Figure 2. Overall, the velocity fields were consistent between control and test valves. The peak velocities of the test valves during systole had values similar to those of the control valves, as shown in Supplemental Table 2. High-speed PIV flow characteristics are shown in Video 2 (23-mm control valve) and Video 3 (23-mm test valve).

## Comment

Structural integrity and hemodynamic stability of bioprosthetic heart valves have been essential measurements to determine the functionality of the valves. Failure to maintain structural integrity can result in leaflet tear, which can cause significant regurgitation. Failure to maintain hemodynamic stability has many clinical implications. Therefore, it is crucial that the valves are tested for mechanical wear to minimize these risks.

In this study, we successfully tested 8 valves (2 control valves and 6 test valves) of sizes 21-mm and 23-mm Inspiris RESILIA valves. The objective was to compare the Inspiris RESILIA valves for fluid mechanic and hemodynamic parameters after very long-term durability studies (equivalent to 50 years in vivo) with their respective control valves (0 cycled). Additionally, the study also aimed to compare the results to historical data (completed with 1 billion cycled valves) and therefore assess the hemodynamics and turbulence characteristics with 2 billion cycled valves.

The Inspiris RESILIA aortic valve showed good structural integrity through the 2 billion cycles of AWT. Both the 0-cycled and 2 billion–cycled valves showed excellent pressure gradients and EOA values for both valve sizes. No extreme leaflet tear was observed in the test valves, and the regurgitant fraction remained consistent with that of the control group. Pressure gradients remained similar, indicating the valve did not have significant changes that can adversely affect the regulation of transvalvular pressures. EOAs were seen to increase slightly in both sizes; this can be attributed to the lengthening or increasing elasticity of the leaflet material over continuous cyclic loading.

Additionally, GOA, as reported in Figure 1 and Supplemental Table E1, was overall higher in the test valves. Quite remarkably, a similar trend was observed in our previous study, where the valves were tested for 1 billion cycles in the AWT.^10^ These results are correspond to the higher EOA in the test group and can be attributed to increased elasticity of the leaflet material over the continuous cyclic loading.

Overall, in both sizes, the valve’s durability, hemodynamics, and kinematics were functionally equivalent to those of the 0-cycled control valves after 2 billion cycles of testing.

There were a few limitations to the study. First, rigid, idealized flow chambers were used. Similarly, the AWT system used for this study is idealized. Second, saline solution was used in the AWT studies. This is an approved medium for testing prosthetic heart valves; however, it is not viscosity matched to blood, and hence results may vary from those using a blood analogue fluid. However, given that flow waveforms are not compared between AWT and pulse duplicator results, the changes can be neglected. Third, the fluid was maintained at room temperature. Temperature changes can cause changes in viscosity, and we can anticipate changes in flow features. However, because only bulk flow features were studied, these minor changes can be neglected. Finally, SVD and hemodynamic stability are multifactorial, and the study did not account for biologic phenomena. This shortcoming of AWT needs to be addressed in the future to allow more realistic evaluation of valve durability. Despite these limitations, our data showed that the Inspiris RESILIA aortic valve is durable for an equivalent of 50 years in the AWT, with findings comparable to values from the long-term mechanical durability study (1 billion cycle study). The biologic advantages of the RESILIA technology and the excellent mechanical durability of the valve could offer significant advantages to reduce long-term calcification in patients.
