# Supplementary material for: Long-term Mechanical Durability of Inspiris RESILIA Surgical Aortic Valves
Source: Ann Thorac Surg Short Rep. 2024 Dec 16;3(2):373–8. doi: 10.1016/j.atssr.2024.11.013 (PMC12167535; doi:10.1016/j.atssr.2024.11.013)
Supplement: Supplemental Material [file mmc1.docx]

**Supplementary Information**

**Methods**

## Particle Image Velocimetry (PIV) Setup

The current study focused on quantitative evaluations of flow fields in the vicinity of the downstream valve using PIV. The valves were mounted on the left heart simulator described in Section ‎2.2 and the PIV setup was tuned as described previously^20,27^. **Figure 1** shows the schematic of the laser plane and the region of interest.

## Data Analysis

Processed PIV images provide velocity vector fields in the plane of interest. The system used in the present experiments was a two-component PIV system, which provided two components of velocity within the plane of interest. After data acquisition, all post-processing was conducted using DaVis 8.4 (LaVision, Göttingen, Germany) and Tecplot 360 (Regenstauf, Germany). Additional derived quantities were calculated from the velocity fields.

By subtracting the mean velocity field from the instantaneous velocity field, a fluctuating velocity field was obtained. This is mathematically represented as:

$u^{'}=U-\bar{U}$ **(eqn 1)**

Where U ̅ is the fluctuating velocity, U is the instantaneous velocity and $u^{'}$ is the mean velocity. The fluctuating velocity field characterizes the levels of variation in the flow fields from cycle to cycle.

Principal Reynolds shear stress (RSS) has been well correlated to blood cell damage and is used to predict potential regions of hemolysis. The principal Reynolds shear stress was calculated for each valve, and is defined as:

$RSS=\rho\sqrt{\left( \frac{\bar{u'u'}-\bar{v'v'}}{2} \right)^{2}+\left( \bar{u'v'} \right)^{2}}$ (**eqn 2)**

**Figures**


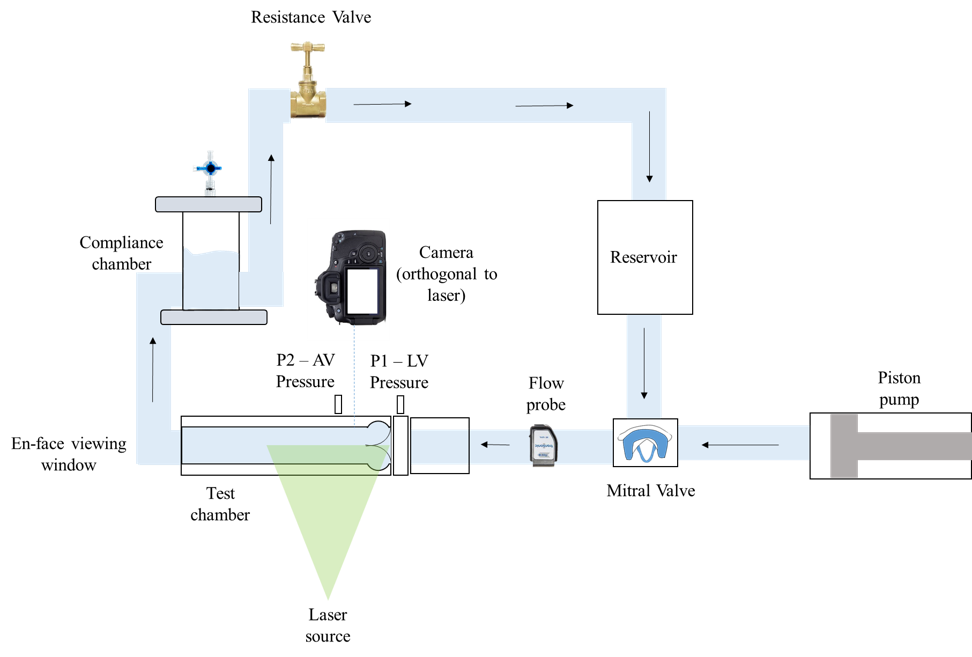


**Figure E1:** Schematic of Georgia Tech Left Heart Simulator with PIV setup


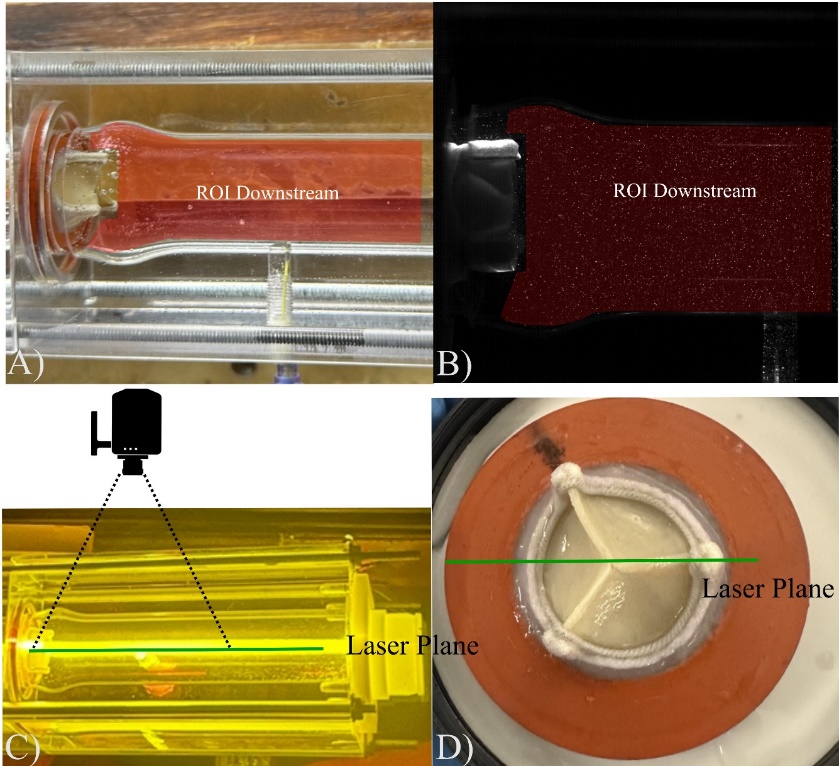


**Figure E2:** Photo illustration of A) ROI of downstream (top view) B) Laser sheet during PIV acquisition (side view), C) Laser plane illustration through valve commissure and D) ROI of downstream in raw

**Tables**

**Table E1:** Geometric orifice area (GOA) integrals for the 21-mm and 23-mm Inspiris RESILIA and Magna Ease valves at zero and 2 billion cycles

| **Valve type** | **0 Cycle** | **2 Billion Cycles** |
| --- | --- | --- |
| 21_Inspiris | 0.39 | 0.37 ± 0.01 |
| 23_Inspiris | 0.42 | 0.50 ± 0.03 |

***Table E2:*** *Max velocities of* *21mm control and test valves during acceleration, peak systole, and deceleration.*

|  | 21 Inspiris RESILIA | | | | 23 Inspiris RESILIA | | | |
| --- | --- | --- | --- | --- | --- | --- | --- | --- |
|  | Control | Test 1 | Test 2 | Test 3 | Control | Test 1 | Test 2 | Test 3 |
| Acceleration | 2.22 | 2.36 | 2.02 | 2.36 | 1.78 | 1.74 | 1.50 | 1.82 |
| Peak Systole | 3.43 | 3.45 | 3.16 | 3.55 | 3.12 | 3.08 | 2.90 | 2.81 |
| Deceleration | 2.21 | 2.25 | 2.12 | 2.16 | 1.97 | 1.95 | 1.83 | 1.76 |
